# Efforts on Changing Lifestyle Behaviors May Not Be Enough to Improve Health-Related Quality of Life Among Adolescents: A Cluster-Randomized Controlled Trial

**DOI:** 10.3389/fpsyg.2021.614628

**Published:** 2021-02-18

**Authors:** Alexsandra da Silva Bandeira, Michael W. Beets, Pablo Magno da Silveira, Marcus Vinicius Veber Lopes, Valter Cordeiro Barbosa Filho, Bruno G. G. da Costa, Kelly Samara Silva

**Affiliations:** ^1^Research Group in Physical Activity and Health, Federal University of Santa Catarina, Department of Physical Education, Florianopolis, Brazil; ^2^Arnold School of Public Health, University of South Carolina, Columbia, SC, United States; ^3^Federal Institute of Education, Science and Technology of Ceara, Aracati Campus, Aracati, Brazil

**Keywords:** physical activity, sedentary behavior, health status, clinical trial, school-based intervention, students

## Abstract

Schools have been the main context for physical activity (PA) and sedentary behavior (SB) interventions among adolescents, but there is inconsistent evidence on whether they also improve dimensions of the health−related quality of life (HRQoL). The aim of this study was to evaluate the effects of a school-based active lifestyle intervention on dimensions of HRQoL. A secondary aim was to verify whether sex, age, and HRQoL at baseline were moderators of the intervention effect. A cluster-randomized controlled trial was conducted at three control and three intervention schools in Florianopolis, Brazil. All students from 7th to 9th grade were invited to participate. A school year intervention, designed primarily to increase PA and reduce SB, included strategies focused on (i) teacher training on PA, SB, and nutrition, and availability of teaching materials related to these contents; (ii) environmental improvements (i.e., creation and revitalization of spaces for the practice of PA in school); and (iii) education strategies, with the availability of folders and posters regarding PA, SB, and nutrition. Participants and the research staffs were not blinded to group assignment, but a standardized evaluation protocol was applied at baseline and after the intervention (March and November 2017) using the KIDSCREEN−27 to assess HRQoL across five dimensions. Mixed linear models were performed to evaluate the effect of the *Movimente* intervention on the five HRQoL dimensions. Of the 921 students who answered the questionnaire at baseline, 300 and 434 completed the study in control and intervention groups, respectively (dropouts: 20%). The results revealed no significant effects of the intervention on any HRQoL dimensions. A reduction of the school environment dimension was observed in both the control (-2.44; 95% CI: -3.41 to -1.48) and intervention groups (-2.09; 95% CI: -2.89 to -1.30). Sensitivity analyses showed that students in the highest baseline tertiles of HRQoL in any dimension had a reduction in their respective scores from pre- to post-intervention in both school groups. In conclusion, our results demonstrated no intervention effect on HRQoL dimensions and those students with the highest levels of HRQoL at baseline on all dimensions reduced from pre to post-intervention.

**Clinical Trial Registration:**

The trial is registered at the Clinical Trial Registry (Trial ID: NCT02944318; date of registration: October 18, 2016).

## Introduction

Health-related quality of life (HRQoL) is defined as a construct that measures global well-being, encompassing the physical, emotional, mental, social, and behavioral domains (Ravens-Sieberer et al., 2006). Although adolescents generally perceived a good health, previous longitudinal studies have found that total HRQoL decreases throughout adolescence (Esteban-Gonzalo et al., 2019; Langeland et al., 2019; Sánchez-Oliva et al., 2019). Therefore, there is a need to develop strategies to sustain and improve HRQoL throughout adolescence (Wu et al., 2017; Marker et al., 2018).

Evidence has shown that physical activity (PA) and sedentary behavior (SB) are associated with HRQoL among adolescents (Jalali-Farahani et al., 2016; Wong et al., 2017; Lee et al., 2019). For instance, after two years of follow-up, Sánchez-Oliva and colleagues found that adolescents who increased their SB had a greater decrease in HRQoL compared to those who changed to active profiles (Sánchez-Oliva et al., 2019). Thus, the development of school-based interventions to promote active lifestyles may be an effective way of improving the HRQoL of adolescents (Wu et al., 2017).

Few studies have examined the effect of the interventions on the HRQoL among healthy adolescents, with the results of existing studies inconsistent (Wu et al., 2017; Marker et al., 2018). A systematic review observed that only three out of thirty-one studies assessed the effect of the school-based PA interventions on adolescents’ HRQoL (Wu et al., 2017). In addition, a recent meta-analysis found that PA interventions positively impact HRQoL, but of the nineteen intervention studies included, only four were developed with apparently healthy children and adolescents (Marker et al., 2018). When considered these PA school-based interventions individually, the results have found inconsistent effects on specific dimensions of HRQoL (Hartmann et al., 2010; Azevedo et al., 2014; Casey et al., 2014; Ha et al., 2015). For instance, PA strategies that were incorporated into physical education classes, and linked to PA opportunities outside school had a positive effect on the physical well-being of Australian girls (Casey et al., 2014). On the other hand, an intervention involving strategies of teacher training on rope skipping, accessibility of resources, and active school recess was not effective for increasing physical well-being, but improved the autonomy and parent’s relation among adolescents of Hong Kong (Ha et al., 2015). In addition, interventions to promote PA and reduce SB at schools, such as providing dance mat systems (Azevedo et al., 2014), additional physical education classes, and breaks during academic lessons (Hartmann et al., 2010; Casey et al., 2014), have presented a positive effect on the psychological well-being of adolescents. The effect of school-based interventions on the dimensions of peers and social support and school environment has been seldom studied, and no effects on these HRQoL dimensions have been observed (Azevedo et al., 2014; Ha et al., 2015). Therefore, the effect of PA and SB interventions in the specific dimensions of adolescents’ HRQoL still requires scientific investigation.

Furthermore, it is important to examine individual characteristics that may influence changes in HRQoL (Ravens-Sieberer et al., 2006; Casey et al., 2014; Meade and Dowswell, 2016; Esteban-Gonzalo et al., 2019), which may change the direction and/or strength (i.e., moderator variable) of the intervention’ effects. Researchers have proposed interventions that seem to be effective “for all” without testing whether the changes in the outcomes occurred in all relevant subgroups of adolescents. For instance, HRQoL can differ according to sex (Meade and Dowswell, 2016; Esteban-Gonzalo et al., 2019), age (Meade and Dowswell, 2016), and preceding HRQoL (Hartmann et al., 2010; Casey et al., 2014). Evidence have found a sex difference in the change over time in HRQoL, with girls being more prone to a reduction in HRQoL scores (Meade and Dowswell, 2016; Esteban-Gonzalo et al., 2019). It was also observed that the younger adolescents presented significantly higher scores of HRQoL throughout three academic years compared to older adolescents (Meade and Dowswell, 2016). In addition, previous findings suggest that adolescents with lower baseline HRQoL scores may be more susceptible to positive changes after an intervention (Ravens-Sieberer et al., 2006; Hartmann et al., 2010; Casey et al., 2014). A study filling these literature gaps may also help researchers and practitioners to identify groups of students that should be focused during school-based interventions that are aiming to improve adolescents’ lifestyle and HRQoL.

To overcome this existing gap in research, we conducted a multicomponent school-based intervention (*Movimente* program) that targeted PA and SB among Brazilian students; additional information, and the results on other behaviors are available online^1^ and in previous publication (Silva et al., 2020). In the present study, we evaluated the effects of this intervention on five dimensions of HRQoL and whether sex, age, and HRQoL at baseline were moderators of the intervention effect among adolescents. We hypothesized that the intervention would positively influence the dimensions of physical well-being, peers and social support, autonomy, and parent’s relation and these effects would differ according to sex, age, and the baseline scores of HRQoL.

## Methods

### Trial Design and Participants

The *Movimente* Program (the Portuguese word for movement) is a cluster randomized controlled trial, with randomization performed at the elementary school level. A detailed description of the theoretical background and methodological approach is in a previous study (Silva et al., 2020). The program was conducted over one school year (March–November 2017). The primary outcomes of the intervention were PA and SB, and secondary outcomes included the five dimensions of HRQoL. The study protocol was approved by the Research Ethics Committee (No:1,259,910, CAAE: 49462015.0.0000.0121; date: November 23, 2015) and the project was registered in the Clinical Trials database (NCT02944318).

**FIGURE 1:**
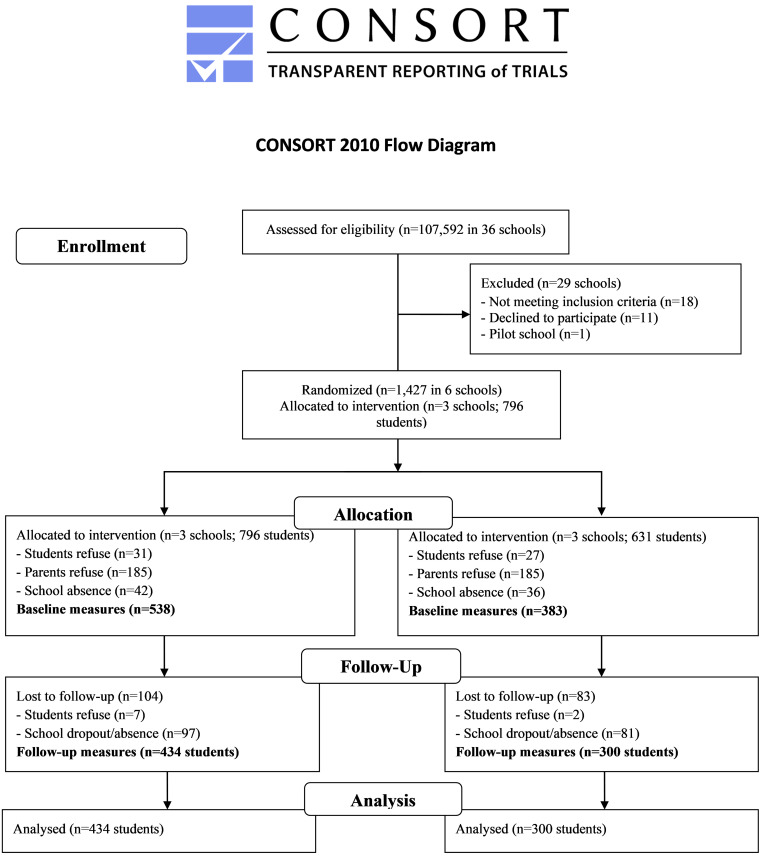
CONSORT flow diagram of recruitment, randomization, and participation of schools and adolescents in the *Movimente* study.

After approval by the Board of Education of the city of Florianopolis (southern Brazil) and recruitment of schools took place in October to November 2015, the inclusion criteria of the schools were: (a) having elementary school (*n* = 27); (b) have at least two classes per grade from the 7th to 9th grade (*n* = 21); and (c) the school could not be undergoing repair works during the collection period (*n* = 18). The 18 schools that met the criteria were invited to participate. Seven schools accepted the invitation, one was selected for the pilot study, in which the strategies were previously tested, and the other six schools were randomly allocated into control and intervention groups, matched by size (two medium schools and one small in each group). All students in grades 7th to 9th from the six selected schools who attended the first weeks of school (1,427 students) were eligible to be part of the program (intervention = 796 and control group = 631). Exclusion criteria for students were: (a) being mentally and/or physically disabled; (b) absence during the first three weeks of the school year. Adolescents with disabilities were included and had the possibility to benefit from the intervention program, as well as the other students. However, we excluded them only from the analyzes as the applied instruments were not adequate to this specific subgroup. Students and parents were asked to sign an assent and consent form, and received no incentive to participate in the intervention. Further details regarding all the intervention procedures can be found in the protocol report (Silva et al., 2020) and Figure 1.

### Intervention

The primary outcomes of the *Movimente* program were the time of total PA and time spent in SB, specifically screen time. Therefore, the strategies were focused to improve these outcomes and they comprised three main components: teacher training, active opportunities in the school environment, and health education for the school community.

Teacher training was specifically designed to provide logistic support to teachers of general disciplines and physical education, and encourage them to talk about health with their students. This strategy was organized in three stages: (i) a 4-h face-to-face meeting, developed at school, to discuss on health issues, PA and SB; (ii) a logistic support via online platform to disclose and discuss the activities developed by teachers at classroom; (iii) a face-to-face 2-h meeting, developed at the end of the intervention, to discuss barriers, facilitators, and intention to continue using activities in their routine. The second component were active opportunities that were promoted in the school environment with the creation and revitalization of spaces for the practice of PA in the school. Each school received a kit of sports equipment (e.g., rackets, skipping rope, balls to play basketball, soccer, volleyball) to make available for students during their free time at school. Finally, health education materials were provided to the school community about the following themes: PA, SB, the relation between PA and academic performance, and eating habits. Every two months, teachers received folders to be discussed in the classroom, and to hand over to the students, which in turn should be delivered to their parents. In addition, at the beginning of the intervention, the school principals received four posters to be placed on school murals.

The strategies were expected to impact physical well-being, as the three components aimed to encourage students to improve PA and reduce SB, and consequently, improve how well and physically fit the student felt. Moreover, it was expected that there would be an effect on the dimension of peers and social support, since the strategies highlighted the importance of social relations with friends, as well as stimulated teachers to provide social support related to PA practice. For instance, teachers were encouraged to develop classroom activities with the educational materials of the intervention, which contained messages that encouraged the relationship between friends (e.g., *The practice of PA can provide opportunities for talks with your friends*; *Reduce time on screens can provide more time with your friends*). Regarding the autonomy and parent relation, some strategies, mainly in the educational component, highlighted the importance of the interactions between parents/family and students, as well as how parents could support their child/adolescent to become more physically active. Despite a lower intensity, it was also expected that the strategies could have an effect on the dimensions of psychological well-being and school environment, since the teachers were encouraged to discuss some psychosocial determinants of PA and SB, as well as there was the availability of new spaces and equipment for PA practice. Due to strategies focused on disseminate information regarding healthier changes on lifestyle and social interactions, positive effects on HRQoL dimensions may emerge independently of changes on PA and SB. Details of strategies of the *Movimente* Program are provided in Supplementary Material, and in previous publication (Silva et al., 2020).

The strategies were developed to reach all eligible students of the intervention schools, independently of their characteristics as sex, age, or level of HRQoL at baseline. Students of control schools continued with their traditional schedule, which included physical education classes twice a week. After the intervention period, the control schools received all materials of the intervention. Additionally, a mixed-method evaluation was conducted to analyze the implementation of the three program components in the intervention schools. In summary, this mixed-method evaluation was conducted considering both qualitative and quantitative measures of the intervention students, as well as their teachers and parents at follow-up. A detailed description of the implementation evaluation will be performed furthermore.

The theoretical basis for the development of the intervention, followed the structure of the program schools promoting health (HPS; Langford et al., 2014) and the theories of behavior change: (i) socio-ecological (Sallis et al., 2006), (ii) social-cognitive (Bandura, 2004), and (iii) transtheoretical (Marcus and Simkin, 1994).

### Outcome Measures

Pre-intervention baseline (March/April 2017) and post-intervention (November/December 2017) measurements occurred at school during class hours. Students answered a standardized questionnaire which was guided and explained by a trained researcher, with two other researchers in the room to support students.

### Health-Related Quality of Life

The HRQoL was measured using Kidscreen-27. This instrument was developed in a large project in 13 European countries, showing Intraclass Correlation Coefficients (ICC) ranging between 0.61 and 0.74 (Ravens-Sieberer et al., 2006). The instrument comprises five dimensions of HRQoL: Physical Well-Being (*n* = 5), Psychological Well-Being (*n* = 7), Autonomy & Parent Relation (*n* = 7), Peers & Social Support (*n* = 4) and School Environment (*n* = 4), the instrument has 27 questions with five response options, according to intensity (not at all, slightly, moderately, very, extremely) or frequency (never, almost never, sometimes, almost always, always). The scores for each dimension are reported as t-values, ranging from 0 to 100. Higher scores indicate better HRQoL (Ravens-Sieberer et al., 2016). Reliability was tested in the pilot study of the *Movimente* program and the Intraclass Correlation Coefficients were observed to range from 0.71 to 0.78 between HRQoL dimensions (Silva et al., 2020).

### Moderators and Covariates

Students reported their sex (male or female), age (completed years), grade (seventh to nineth), and answered to a checklist of the ownership of household items according to the Brazilian Economic Classification Criteria (e.g., number of cars, refrigerators, and computers). These items were used to estimate an asset index by applying Principal Component Analysis, which is a proxy of socioeconomic status (SES; Vyas and Kumaranayake, 2006). The SES score ranged from 0 to 15, with higher values referring to greater family wealth. In addition, as there is no classification recommended by the Kidscreen group regarding HRQoL scores, baseline terciles values of HRQoL dimensions were considered for analysis.

### Analyses

Sample characteristics were described using mean and standard deviation for continuous variable, and absolute and relative frequency for categorical variables. Student’s *t*-tests and Pearson’s chi-squared tests were applied to compare control and intervention groups at baseline, and to compare the baseline sample with dropouts.

Three-level linear mixed models were performed to evaluate the effect of the *Movimente* intervention on the five HRQoL dimensions. All models considered repeated measures (pre- and post-intervention) nested within participants, which were nested within schools. This hierarchical structure was used to consider the sampling design and the clustered nature of the data. As mixed models can accommodate unbalanced data, all available measures were included in analysis. The interaction term of condition (intervention vs control) by time (post- vs pre-intervention) and the time invariant covariates (i.e., sex, grade, and SES) were included as fixed effects. Sensitivity analyses were performed to evaluate potential moderators of the interventions’ effect by testing tree-way interactions terms (condition × time × moderator). The variables sex (Meade and Dowswell, 2016; Esteban-Gonzalo et al., 2019), age (Meade and Dowswell, 2016), and baseline terciles of HRQoL dimensions (Hartmann et al., 2010; Casey et al., 2014) were tested as potential moderators based on previous evidence. Thus, the slopes of time by group were computed for each level of the moderator variable. Fitted models were evaluated according to the assumptions of homoscedasticity and residuals normality. Conclusions on interventions effect was conducted by comparing 95% confidence intervals of the post-pre mean differences between intervention and control groups. Statistical analyses were conducted in Stata, version 14.0 (StataCorp LP., College Station, TX, United States).

## Results

### Recruitment and Baseline Measures

Six out of 18 eligible schools indicated interest in the study, and three were randomly assigned for each condition. Of the 1,427 eligible students (control: 631; intervention: 796), 921 students participated of the baseline measures (control: 383; intervention: 538), and 734 (control: 300; intervention: 434) completed the study (dropouts: 20%). At baseline, 370 adolescents did not deliver the consent form, 58 refused to participate in the *Movimente* Program, and 78 were absent from school. At follow-up, a total of 187 adolescents were not assessed: 178 because of being absent, and nine students refused to participate.

At baseline, the majority of students were girls (control: 52.7%; intervention: 51.1%), and students aged from 10 to 13 years old (control: 60.9%; intervention: 64.7%) (Table 1). The dimensions of HRQoL ranged from 43.8 (±10.0, physical) to 49.6 (±10.5, peers) for control schools, and 44.1 (±9.7, physical) to 49.8 (±10.3, peers) for intervention schools. There were no differences between intervention and control schools in any variables at baseline. Dropouts were older, and presented lower scores of HRQoL than participants who completed the trial (*p* < 0.05, Table 1).

**TABLE 1:** Students’ characteristics at baseline according to group (intervention and control) and in participants and dropouts of the *Movimente* Program, 2017.

**Sociodemographic variables**	**Participants (*n* = 734)**		**Dropouts (*n* = 187)**		***p*-value**	**Control (*n* = 383)**		**Intervention (*n* = 538)**		***p*-value**
**Sex**	*n* (%)		*n* (%)			*n* (%)		*n* (%)		
Boys	356 (48.5)		88 (47.1)		0.725	181 (47.1)		263 (48.9)		0.626
Girls	378 (51.5)		99 (52.9)			202 (52.7)		275 (51.1)		
**Age**										
10 to 13	477 (65.1)		103 (55.4)		0.014	232 (60.9)		348 (64.7)		0.241
14 to 16	256 (34.9)		83 (44.6)			149 (39.1)		190 (35.3)		
**SES** (mean ± SD)	4.9 (1.8)		4.8 (1.8)		0.507	4.9 (1.9)		4.9 (1.8)		0.867

**HRQoL**	***n***	**Mean (± SD)**	***n***	**Mean (± SD)**		***n***	**Mean (± SD)**	**n**	**Mean (± SD)**	

Physical	727	44.3 (10.1)	185	42.4 (8.7)	0.018	378	43.8 (10.0)	534	44.1 (9.7)	0.748
Psychological	720	46.1 (11.4)	182	43.9 (11.8)	0.017	372	45.6 (12.5)	530	45.8 (10.8)	0.864
Autonomy and Parent’s relation	724	47.1 (8.9)	179	45.4 (9.9)	0.035	372	46.5 (9.9)	531	46.9 (8.5)	0.558
Peers and Social support	727	50.3 (10.2)	185	47.3 (10.6)	< 0.001	378	49.6 (10.5)	534	49.8 (10.3)	0.721
School	725	48.8 (9.0)	183	46.3 (8.9)	0.004	377	48.3 (9.4)	531	48.4 (8.6)	0.946

*P-value estimated using the Chi-square test for categorical variables and independent *t*-test for continuous variables; Socioeconomic Status (SES) was the variable with the highest missing data (*n* = 86).*

### Intervention Effects

**FIGURE 2:**
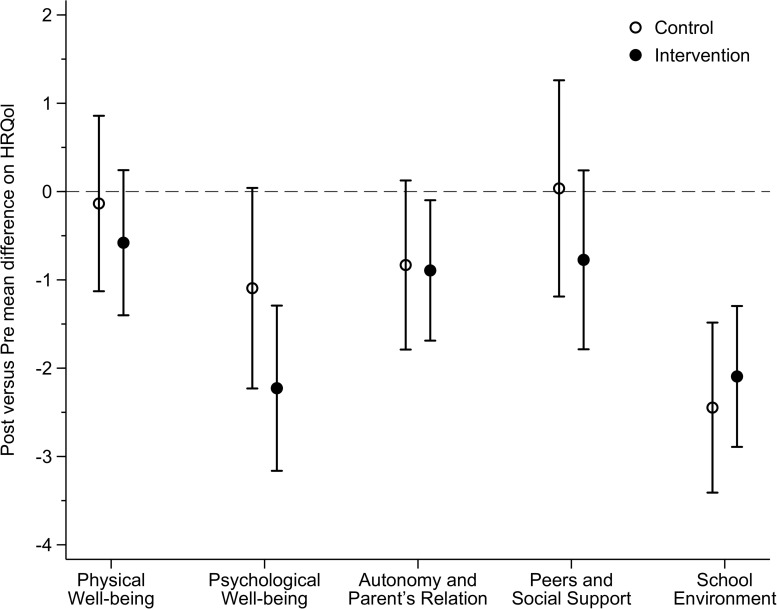
Effect of the Movimente Program on dimensions of HRQoL among total sample.

**FIGURE 3:**
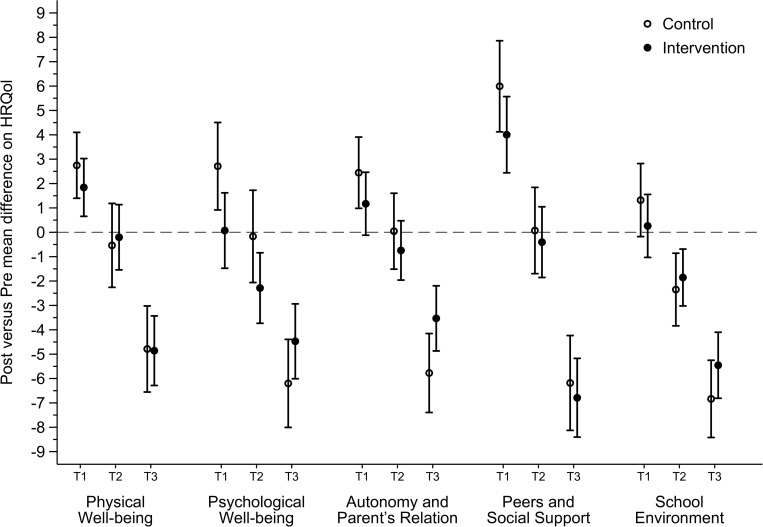
Effect of the Movimente Program on dimensions of HRQoL according the terciles (T1: lower; T2: medium; T3: highest) of HRQoL at baseline.

As shown in Table 2 and Figure 2, the adjusted mean of the school dimension reduced for both control group (-2.45; 95% CI: -3.41 to -1.48; effect size: -0.26) as for the intervention group (-2.09; 95% CI: -2.89 to -1.30; effect size: -0.22), but there was no difference between groups. Sensitivity analyses showed that students of the highest baseline terciles of all HRQoL dimensions reduced their HRQoL scores from pre- to post-intervention in both control and intervention groups (Figure 3 and Supplementary Material). However, time (pre-post difference) by condition (intervention or control group) interaction effects did not vary between sexes, age groups and terciles of baseline HRQoL.

**TABLE 2:** Effects of the *Movimente* Program on dimensions of HRQoL among total sample.

**Dimensions**	**Estimates**				
	**Baseline mean**	**Follow-up mean**	**β (95% CI)**	**Delta (%)**	**Effect Size**
**Physical well-being**					
Control	44.1	44.0	−0.14 (−1.13,0.86)	−0.31	−0.01
Intervention	44.1	43.5	−0.58 (−1.40,0.24)	−1.31	−0.06
**Psychological well-being**					
Control	46.0	45.0	−1.09 (−2.23,0.04)	−2.38	−0.09
Intervention	45.7	43.5	−2.23 (−3.16, −1.29)	−4.87	−0.19
**Autonomy and parent’s relation**					
Control	46.9	46.1	−0.83 (−1.79,0.13)	−1.77	−0.09
Intervention	47.0	46.1	−0.89 (−1.69, −0.10)	−1.90	−0.10
**Peers and social support**					
Control	49.9	50.0	0.04 (−1.19,1.26)	0.07	0.00
Intervention	49.9	49.1	−0.77 (−1.79,0.24)	−1.55	−0.07
**School environment**					
Control	48.6	46.2	−2.45 (−3.41, −1.48)	−5.03	−0.26
Intervention	48.5	46.4	−2.09 (−2.89, −1.30)	−4.32	−0.22

*Linear mixed models were used to calculate baseline and follow-up means, adjusted for sex, age, grade, SES, and HRQoL.*

## Discussion

We hypothesized that our school-based active lifestyle intervention would positively influence the HRQoL dimensions of physical well-being, peers and social support, as well as the autonomy and parent’s relation; and these effects would differ according to sex, age, and the previous level of HRQoL. However, our analyses revealed no significant effects of the intervention on any HRQoL dimensions, but we found that the level of HRQoL at baseline was a moderator for the pre-post changes in all dimensions among both control and intervention conditions. Previous school-based interventions have found inconsistent evidence about effects on HRQoL (Hartmann et al., 2010; Finkelstein et al., 2013; Azevedo et al., 2014; Casey et al., 2014; Quaresma et al., 2014; Ha et al., 2015), which suggests that, owing to the multidimensionality of HRQoL, strategies to promote PA and reduce SB may not be enough to generate significant changes in the dimensions of HRQoL among adolescents.

Our results were distinct from a previous meta-analysis, which showed that PA interventions had a positive effect on global HRQoL; however, it included studies with healthy and chronic illness adolescents (Marker et al., 2018). When considered intervention effects on different HRQoL dimensions of healthy adolescents, findings are not consistent (Hartmann et al., 2010; Finkelstein et al., 2013; Azevedo et al., 2014; Casey et al., 2014; Quaresma et al., 2014; Ha et al., 2015). Regarding the physical well-being dimension of HRQoL, Casey et al. (2014) found that PA strategies for girls, into physical education classes and linked to PA opportunities outside school, provided positive effects, preventing the reduction of this dimension in the intervention group. However, studies combining both sexes have not shown significant effects on the physical well-being dimension of HRQoL (Hartmann et al., 2010; Finkelstein et al., 2013; Azevedo et al., 2014; Ha et al., 2015). A possible explanation for these results is that interventions did not have significant effects on the PA outcomes and, consequently, it was not able to improve the physical HRQoL of adolescents (Azevedo et al., 2014; Ha et al., 2015). Moreover, it is possible that school-based interventions may not have significant effects on healthy adolescents, or in subgroups that are less susceptible to reduce this dimension over time, since they already evaluate themselves with good physical HRQoL, which may mean that meaningful improvement is harder to achieve or that changes may be perceived in the long term from sustained chronic effects (Hartmann et al., 2010).

According our knowledge, only two previous studies evaluated the effects of PA interventions on peers and social support dimension among healthy adolescents, and both found no significant effects (Azevedo et al., 2014; Ha et al., 2015), which are aligned with our findings. On the other hand, the authors showed that strategies, including rope skipping programs and dance mat systems, were able to positively change the dimension of autonomy and parent’s relation (Azevedo et al., 2014; Ha et al., 2015). Possibly, the lack of effect on these two HRQoL dimensions may be related to some barriers that prevented the successful implementation of the program strategies. Despite the fact that the present intervention included strategies aimed to improve the parents’ and peers’ support for PA, the evaluation of the program’s implementation found little engagement by parents and students in developing the strategies (data not shown). Furthermore, the dimension of autonomy and parent’s relation also considers aspects regarding the perceived autonomy of the adolescents, including financial. Considering that HRQoL was not a primary outcome of the *Movimente* program, specific strategies for improving different types of autonomy were not considered. Therefore, future interventions that establish HRQoL as primary outcome may consider these dimensions for development of intervention strategies, aiming to make adolescents more autonomous, as well as to consider aspects that improve the implementation of strategies. The involvement of parents and friends, and the implementation of specific PA such as sports, dance, and others for boys and girls may also improve the effectiveness of interventions, as preference for PA types may differ according to sex (Bertuol et al., 2020), as well as its relation with HRQoL dimensions (Costa et al., 2020).

Considering the possible moderators, adolescents with higher baseline HRQoL had a reduction in their respective outcomes in both intervention and control groups. To our knowledge, no studies evaluated baseline HRQoL level as a possible moderator of the effect of the intervention on HRQoL dimensions. A possible explanation for our results is the context in which students lived at the time of data collection. The post-intervention measurement coincided with the end of the school year, with academic assessments that may have negatively influenced the HRQoL of adolescents (García-Moya et al., 2019). When considered intervention effects on HRQoL dimensions according sex and age groups, no significant difference was found between groups. Previous findings suggest that girls and older adolescents were more susceptible to present less HRQoL scores when compared with their peers (Meade and Dowswell, 2016; Esteban-Gonzalo et al., 2019), which indicates these two subgroups as more likely to improve HRQoL scores (Ravens-Sieberer et al., 2006; Hartmann et al., 2010; Casey et al., 2014). However, our intervention has not confirmed these hypotheses, thus it is important that future studies to evaluate which groups of adolescents that should be focused during school-based interventions aiming to improve HRQoL.

Collaborating with this finding, there was also a reduction on school environment dimension in both intervention and control groups. Two previous interventions that evaluated the effects of PA strategies on school environment dimension found no significant effects (Azevedo et al., 2014; Ha et al., 2015). However, a comparison with our results should be made carefully because studies were different in the types of intervention strategies used and the social context of schools. Probably, this reduction occurs because the dimension of school environment explores the perception of the student about their cognitive ability, learning, and concentration, which may be negatively influenced by the end of the academic year. For instance, longitudinal evidence has presented a reduction on school environment dimension, as well as an increase on emotional problems among adolescents from final years of elementary school (García-Moya et al., 2019). Also, the authors observed that the school environment dimension is linked to emotional problems in early adolescence (García-Moya et al., 2019), supporting our previous argument. Thus, it is notable that the current evidence highlighted the need for a school environment that provides strategies to promote well-being to students.

### Strengths and Limitations

Although previous studies have evaluated the effect of the interventions on HRQoL, as far as are aware, this is the first school-based intervention exploring the effect of active lifestyle strategies on different dimensions of HRQoL among adolescents from a middle-income country. Moreover, we conducted sensitivity analysis to evaluate the effect of the intervention among potential moderators, an important issue that was previously highlighted, since the strategies could have a different impact depending on the individual characteristics of adolescents. Limitations of the current study should be recognized and addressed in future interventions. First, HRQoL was not the primary outcome of the *Movimente* Program, which may have been related to the non-significant effect on the dimensions. Although our strategies have comprised some aspects of the different dimensions of HRQoL, it seems to have been insufficient to change these outcomes. Finally, we carried out post-intervention measures at the end of the school year, coinciding with the academic assessments that may have negatively influenced the HRQoL of adolescents.

### Implications for Research and Practice

Although we understand that multicomponent interventions are extremely necessary to improve the different aspects of health, the focus on more than one target outcome, and more than one theory in the same intervention may be complex to obtain substantial changes in the outcomes. Therefore, future interventions to improve HRQoL may focus on specific strategies for its dimensions, with the possibility of evaluating different arms of interventions to compare scenarios and strategies focused on each dimension. In this regard, behavioral theories are important tools in the planning and implementation of these strategies, and how the changes on lifestyle behaviors may help HRQoL dimensions. In addition, it is important to consider the different levels of HRQoL of students since they tend to respond in different ways. Finally, considering that there is a tendency to reduce the dimensions of HRQoL over time, the school community must be careful in maintaining a healthy school environment that provides psychological well-being to students during the school year.

## Conclusion

The *Movimente* Program had no effect on HRQoL dimensions, and we observed that the level of HRQoL reduced from pre to post-intervention among those with higher HRQoL at the baseline. However, there was also no intervention effect on the changes of HRQoL dimensions, considering the possible moderators.

## Data Availability Statement

The raw data supporting the conclusions of this article is not available due to ethical restrictions.

## Ethics Statement

The studies involving human participants were reviewed and approved by National Research Ethics Committee (No: 1,259,910, CAAE: 49462015.0.0000.0121; date: in November 23rd, 2015). Written informed consent to participate in this study was provided by the participants’ legal guardian/next of kin.

## Author Contributions

AB, PS, ML, and BC carried out the analysis and written the first draft of the manuscript. MB, VB, and KS critically reviewed and commented on previous versions of the manuscript. All authors read, helped to draft and approved the final version of the manuscript.

## Conflict of Interest

The authors declare that the research was conducted in the absence of any commercial or financial relationships that could be construed as a potential conflict of interest.

## Footnotes

1 www.movimente.ufsc.br
