# Supplementary material for: Efforts on Changing Lifestyle Behaviors May Not Be Enough to Improve Health-Related Quality of Life Among Adolescents: A Cluster-Randomized Controlled Trial
Source: Front Psychol. 2021 Feb 18;12:614628. doi: 10.3389/fpsyg.2021.614628 (PMC7929984; doi:10.3389/fpsyg.2021.614628)
Supplement: Supplementary file 1 [file Table_1.docx]

| **Table 1**. Details of the *Movimente* Program | | |
| --- | --- | --- |
| **Component** | **Strategies** | **Relationship between strategies and dimensions HRQoL** |
| Teacher Training | Face-to-face meeting focused on health topics, and to discuss possible activities to perform with students to improve PA and reduce SB. | *Physical Well-Being*: teachers received training about how to include PA content and break activities into the classroom. This strategy aimed stimulates the students to improve PA and reduce SB, and consequently, and improve how well and physically healthy the student feels.  *Psychological Well-Being*: the teachers were stimulated to discuss with students about psychosocial determinants of PA and SB. It may be related to improvement of their positive emotions, as well as confidence.  *Peers & Social Support*: teachers were encouraged to highlight the importance of social relations with friends, as well as providing social support related to PA practice. |
|  | Support material (book) with activities was provided to assist the teachers with several lessons plan about PA and SB. |  |
|  | Interactive media (social media and text messaging apps) for teachers to disclose and discuss their activities regarding to health topics. |  |
| Environmental Improvements | Creation of new spaces | *Physical Well-Being*: the availability of new spaces and equipment may be related to an increase in the practice of PA and, consequently, an improvement in the physical well-being of adolescents.  *Psychological Well-Being*: the diversity of spaces and equipment available could encourage adolescents to have different alternatives for PA practice, to promote positive emotions and inhibit feelings such as loneliness and sadness.  *School Environment*: the availability of new spaces and equipment for PA practice could improve the perception and feelings of students about the environment school. |
|  | Revitalization of some spaces of the school for the practice of PA |  |
|  | PA equipment (balls, jump ropes, rackets, etc.) available to students during free-time in school. |  |
| Educational Actions | Four banners and four folders about PA and health, SB and health, PA and academic performance and eating habits. | *Physical Well-Being:* This strategy aimed to stimulate the students to improve PA and reduce SB, and consequently, and improve how well and physically healthy the student feels.  *Autonomy & Parent Relation:* the messages highlighted the importance of interactions between parents/family and students, as well as how parents could support their child/adolescent to become more physically active.  *Peers & Social Support*: the messages highlighted the importance of social relations with friends (e.g., *The practice of PA can provide opportunities for talks with your friends; Reduce time on screens can provide more time with your friends*) |
|  |  |  |
| AF: Physical Activity; SB: Sedentary Behavior; | | |
