# Supplementary material for: Efforts on Changing Lifestyle Behaviors May Not Be Enough to Improve Health-Related Quality of Life Among Adolescents: A Cluster-Randomized Controlled Trial
Source: Front Psychol. 2021 Feb 18;12:614628. doi: 10.3389/fpsyg.2021.614628 (PMC7929984; doi:10.3389/fpsyg.2021.614628)
Supplement: Supplementary file 2 [file Table_2.docx]

| **Table -** Effect of the *Movimente* Program on dimensions of HRQoL according the sex, age groups, and terciles of HRQoL at baseline. | | | | | | |
| --- | --- | --- | --- | --- | --- | --- |
| **Moderators** | **Physical Well-being** | | | | | |
|  | Control | | | Intervention | | |
|  | 𝛽 (95%CI) | Delta (%) | Effect-size | 𝛽 (95%CI) | Delta (%) | Effect-size |
| **Sex** |  |  |  |  |  |  |
| Male | 0.96 (-0.49,2.40) | 2.06 | 0.10 | 0.43 (-0.75,1.60) | 0.89 | 0.04 |
| Female | -1.09 (-2.44,0.26) | -2.61 | -0.11 | -1.54 (-2.68,-0.40) | -3.75 | -0.15 |
| **Age group** |  |  |  |  |  |  |
| 10 to 13 years old | -0.64 (-1.88,0.59) | -1.40 | -0.06 | -1.10 (-2.11,-0.09) | -2.41 | -0.11 |
| 14 to 16 years old | 0.81 (-0.85,2.47) | 1.98 | 0.08 | 0.49 (-0.92,1.89) | 1.18 | 0.05 |
| **Dimension Terciles** | |  |  |  |  |  |
| 1st tercile | 2.75 (1.40,4.10) | 7.55 | 0.27 | 1.84 (0.66,3.03) | 5.04 | 0.18 |
| 2nd tercile | -0.54 (-2.26,1.19) | -1.20 | -0.05 | -0.20 (-1.54,1.13) | -0.46 | -0.02 |
| 3rd tercile | -4.79 (-6.55,-3.02) | -8.45 | -0.48 | -4.86 (-6.29,-3.43) | -8.78 | -0.48 |
|  | **Psychological Well-being** | | | | | |
|  | Control | | | Intervention | | |
|  | 𝛽 (95%CI) | Delta (%) | Effect-size | 𝛽 (95%CI) | Delta (%) | Effect-size |
| **Sex** |  |  |  |  |  |  |
| Male | -0.39 (-2.04,1.25) | -0.80 | -0.03 | -0.29 (-1.62,1.03) | -0.61 | -0.02 |
| Female | -1.71 (-3.25,-0.17) | -3.96 | -0.14 | -4.07 (-5.36,-2.78) | -9.42 | -0.34 |
| **Age group** |  |  |  |  |  |  |
| 10 to 13 years old | -1.24 (-2.65,0.18) | -2.64 | -0.10 | -2.40 (-3.55,-1.24) | -5.15 | -0.20 |
| 14 to 16 years old | -0.80 (-2.71,1.11) | -1.80 | -0.07 | -1.85 (-3.46,-0.25) | -4.18 | -0.16 |
| **Dimension Terciles** | |  |  |  |  |  |
| 1st tercile | 2.71 (0.92,4.51) | 7.90 | 0.23 | 0.07 (-1.48,1.62) | 0.20 | 0.01 |
| 2nd tercile | -0.17 (-2.06,1.73) | -0.37 | -0.01 | -2.29 (-3.73,-0.84) | -5.07 | -0.19 |
| 3rd tercile | -6.20 (-8.01,-4.39) | -10.62 | -0.52 | -4.47 (-6.01,-2.94) | -7.82 | -0.38 |
|  | **Autonomy and Parent’s Relation** | | | | | |
|  | Control | | | Intervention | | |
|  | 𝛽 (95%CI) | Delta (%) | Effect-size | 𝛽 (95%CI) | Delta (%) | Effect-size |
| **Sex** |  |  |  |  |  |  |
| Male | -0.19 (-1.59,1.20) | -0.41 | -0.02 | 0.28 (-0.85,1.42) | 0.59 | 0.03 |
| Female | -1.38 (-2.68,-0.08) | -2.95 | -0.15 | -2.01 (-3.11,-0.91) | -4.35 | -0.22 |
| **Age group** |  |  |  |  |  |  |
| 10 to 13 years old | -1.41 (-2.60,-0.21) | -2.95 | -0.15 | -1.06 (-2.04,-0.08) | -2.23 | -0.11 |
| 14 to 16 years old | 0.20 (-1.39,1.78) | 0.43 | 0.02 | -0.55 (-1.90,0.81) | -1.19 | -0.06 |
| **Dimension Terciles** | |  |  |  |  |  |
| 1st tercile | 2.44 (0.98,3.91) | 6.34 | 0.26 | 1.17 (-0.12,2.46) | 3.02 | 0.13 |
| 2nd tercile | 0.04 (-1.51,1.60) | 0.10 | 0.00 | -0.74 (-1.96,0.47) | -1.60 | -0.08 |
| 3rd tercile | -5.77 (-7.39,-4.15) | -9.95 | -0.62 | -3.53 (-4.87,-2.20) | -6.22 | -0.38 |
|  | **Peers and Social Support** | | | | | |
|  | Control | | | Intervention | | |
|  | 𝛽 (95%CI) | Delta (%) | Effect-size | 𝛽 (95%CI) | Delta (%) | Effect-size |
| **Sex** |  |  |  |  |  |  |
| Male | 0.12 (-1.66,1.91) | 0.25 | 0.01 | -0.54 (-1.99,0.92) | -1.07 | -0.05 |
| Female | -0.04 (-1.72,1.64) | -0.09 | 0.00 | -1.00 (-2.41,0.41) | -2.00 | -0.09 |
| **Age group** |  |  |  |  |  |  |
| 10 to 13 years old | -0.80 (-2.33,0.73) | -1.58 | -0.08 | -0.77 (-2.02,0.48) | -1.52 | -0.07 |
| 14 to 16 years old | 1.60 (-0.44,3.64) | 3.29 | 0.15 | -0.75 (-2.48,0.98) | -1.54 | -0.07 |
| **Dimension Terciles** | |  |  |  |  |  |
| 1st tercile | 5.99 (4.12,7.86) | 15.48 | 0.57 | 4.00 (2.44,5.57) | 10.27 | 0.38 |
| 2nd tercile | 0.07 (-1.70,1.84) | 0.15 | 0.01 | -0.40 (-1.85,1.05) | -0.81 | -0.04 |
| 3rd tercile | -6.18 (-8.13,-4.24) | -9.94 | -0.59 | -6.79 (-8.40,-5.17) | -10.87 | -0.64 |
|  | **School Environment** | | | | | |
|  | Control | | | Intervention | | |
|  | 𝛽 (95%CI) | Delta (%) | Effect-size | 𝛽 (95%CI) | Delta (%) | Effect-size |
| **Sex** |  |  |  |  |  |  |
| Male | -2.07 (-3.47,-0.66) | -4.26 | -0.22 | -1.28 (-2.42,-0.14) | -2.63 | -0.14 |
| Female | -2.78 (-4.10,-1.46) | -5.71 | -0.30 | -2.87 (-3.99,-1.76) | -5.95 | -0.31 |
| **Age group** |  |  |  |  |  |  |
| 10 to 13 years old | -2.52 (-3.72,-1.31) | -5.11 | -0.27 | -2.51 (-3.49,-1.53) | -5.07 | -0.27 |
| 14 to 16 years old | -2.28 (-3.89,-0.68) | -4.82 | -0.24 | -1.26 (-2.62,0.11) | -2.70 | -0.13 |
| **Dimension Terciles** | |  |  |  |  |  |
| 1st tercile | 1.32 (-0.18,2.82) | 3.38 | 0.14 | 0.26 (-1.03,1.55) | 0.66 | 0.03 |
| 2nd tercile | -2.35 (-3.84,-0.86) | -4.88 | -0.25 | -1.85 (-3.02,-0.69) | -3.85 | -0.20 |
| 3rd tercile | -6.83 (-8.42,-5.25) | -11.35 | -0.73 | -5.45 (-6.81,-4.10) | -9.21 | -0.58 |

1st tercile: lower; 2nd tercile: medium; 3rd tercile: highest
